# acdc – Automated Contamination Detection and Confidence estimation for single-cell genome data

**DOI:** 10.1186/s12859-016-1397-7

**Published:** 2016-12-20

**Authors:** Markus Lux, Jan Krüger, Christian Rinke, Irena Maus, Andreas Schlüter, Tanja Woyke, Alexander Sczyrba, Barbara Hammer

**Affiliations:** 1 Computational Methods for the Analysis of the Diversity and Dynamics of Genomes, Bielefeld University, Universitätsstr. 25, Bielefeld, 33615 Germany; 2 Center for Biotechnology - CeBiTec, Bielefeld University, Universitätsstr. 27, Bielefeld, 33615 Germany; 3 2800 Mitchell Drive, Walnut Creek, 94598 CA USA; 4 CITEC centre of excellence, Bielefeld University, Inspiration 1, Bielefeld, 33619 Germany; 5 Australian Centre for Ecogenomics, University of Queensland, ST LUCIA, Brisbane, QLD 4072 Australia

**Keywords:** Single-cell sequencing, Contamination detection, Machine learning, Clustering, Binning, Quality control

## Abstract

**Background:**

A major obstacle in single-cell sequencing is sample contamination with foreign DNA. To guarantee clean genome assemblies and to prevent the introduction of contamination into public databases, considerable quality control efforts are put into post-sequencing analysis. Contamination screening generally relies on reference-based methods such as database alignment or marker gene search, which limits the set of detectable contaminants to organisms with closely related reference species. As genomic coverage in the tree of life is highly fragmented, there is an urgent need for a reference-free methodology for contaminant identification in sequence data.

**Results:**

We present acdc, a tool specifically developed to aid the quality control process of genomic sequence data. By combining supervised and unsupervised methods, it reliably detects both known and de novo contaminants. First, 16S rRNA gene prediction and the inclusion of ultrafast exact alignment techniques allow sequence classification using existing knowledge from databases. Second, reference-free inspection is enabled by the use of state-of-the-art machine learning techniques that include fast, non-linear dimensionality reduction of oligonucleotide signatures and subsequent clustering algorithms that automatically estimate the number of clusters. The latter also enables the removal of any contaminant, yielding a clean sample. Furthermore, given the data complexity and the ill-posedness of clustering, acdc employs bootstrapping techniques to provide statistically profound confidence values. Tested on a large number of samples from diverse sequencing projects, our software is able to quickly and accurately identify contamination. Results are displayed in an interactive user interface. Acdc can be run from the web as well as a dedicated command line application, which allows easy integration into large sequencing project analysis workflows.

**Conclusions:**

Acdc can reliably detect contamination in single-cell genome data. In addition to database-driven detection, it complements existing tools by its unsupervised techniques, which allow for the detection of de novo contaminants. Our contribution has the potential to drastically reduce the amount of resources put into these processes, particularly in the context of limited availability of reference species. As single-cell genome data continues to grow rapidly, acdc adds to the toolkit of crucial quality assurance tools.

**Electronic supplementary material:**

The online version of this article (doi:10.1186/s12859-016-1397-7) contains supplementary material, which is available to authorized users.

## Background

Modern sequencing technologies provide sample substrate for the analysis of large amounts of genetic information. Specifically, single-cell sequencing (SCS) is now one of the most powerful methods in genome discovery and analysis. Named *Method of the Year 2013* [1], it plays an increasingly important role in many domains. Notable areas of research include medicine and the analysis of disease pathways [2], especially in cancer biology [3] and the development of targeted treatments (personalized medicine) [4]. Additionally, SCS has proven a valuable and very powerful tool in evolutionary and environmental microbiology, for example by assessing intra- and inter-phylum relationships of Bacteria and Archaea [5] and providing insights into key metabolic functions of uncultivated clades within their ecosystems [6].

A primary challenge in single-cell sequence data is the potential presence of contamination and the detection thereof [7]. Foreign DNA which does not belong to the target genome of a given single cell, might be introduced into a sample in different ways. Sources of contamination can include unclean lysis or whole genome amplification reagents, in addition to sample introduced non-target DNA [8, 9].

While much effort has been invested into engineering devices and methods for cell isolation and amplification steps that minimize contamination caused by the surrounding sequencing setup [7, 8, 10], careful quality control is vital to prevent the propagation of misleading results in public databases.

Given those obstacles, ProDeGe, an automated Protocol for the Decontamination of Genomes was recently developed [11]. ProDeGe combines the BLAST algorithm [12] as a popular choice for database sequence alignment with reference-free PCA-reduced oligonucleotide profiling to enhance classification accuracy. Another method, CheckM [13], solely relies on the presence of multiple single-copy marker genes as an indication for contamination in a given sample, not operating reference-free. More recent classification methods [14, 15], most notably Kraken [16], are as accurate as BLAST but much faster, thus can speed up supervised detection. All these techniques heavily rely on references, hence they require existing knowledge about the characteristics of possible contaminants, making them less applicable either in the case of contaminants not being contained in databases or marker genes not being present in the sample (i.e. contamination is small or incomplete). Since the majority of species is unknown [5], they are difficult to detect by such methods and unsupervised, taxonomy-free analysis is required [17].

Complementary to reference-based methods, clustering of oligonucleotide signatures is a promising approach that already found early application in metagenomic binning [18–20]. From the perspective of computational intelligence, contamination detection as a clustering problem is very similar to metagenomic binning. Both metagenomic and SCS samples can be represented as a set of high-dimensional data points. Binning and also contamination detection then address the same challenge of reliably detecting clusters in a high-dimensional data space. In this context, quite a few challenges arise: To circumvent negative side effects in such high-dimensional spaces [21] and to enable human expert inspection, it is crucial to use appropriate subspace embeddings to transform the data into an easily visualizable representation, i.e. two or three dimensions. Modern, non-linear dimensionality reduction methods, in particular Barnes-Hut-SNE (BH-SNE) [22] have proven successful [18, 19] in that context.

The automatic determination of the number of clusters and its cluster validity, a deep and crucial question in the context of clustering [23, 24], poses yet another challenge. In contrast to metagenomic binning where the aim is to accurately bin sequences in a larger number of clusters, contamination detection in SCS requires the discrimination between one or more clusters (genomes). This complication heavily reduces the set of applicable clustering algorithms: The majority of methods for estimating the number of clusters rely on cluster-specific measures such as internal validity indices [25]. Since these are not defined for only one cluster, a distinctive null model for unimodal data is required, i.e. the techniques are usually not suited to distinguish one versus more than one cluster, hence cannot reliably identify non-contaminated samples.

Last, machine learning methods such as dimensionality reduction and clustering are based on statistics of the data and introduce certain amounts of variance. To overcome this limitation and to provide accurate and interpretable results, it is useful to integrate confidence measures. For this task, bootstrapping [26] is a popular choice.

In this contribution, we present a novel software tool called acdc (*Automated Contamination Detection and Confidence estimation for single-cell genome data*), which seamlessly integrates reference-based with reference-free methods. Being based on both, very fast exact database alignments and modern techniques from unsupervised machine learning, acdc is able to accurately identify contamination in single-cell sequencing data. To our knowledge, integrating entirely reference-free methodologies is a novelty, and complements existing high performing database-driven approaches such as ProDeGe. The use of appropriate clustering algorithms allow the removal of foreign sequences to yield clean single-cell genome assemblies. Additionally, the integration of statistically profound confidence values support expert interpretation. As we expect single-cell genomes to further and rapidly populate public databases, acdc will be a resource-effective tool in the quality assurance of single-cell draft genomes, especially for users who do not have the background to use the included techniques directly.

## Implementation

**Fig. 1:**
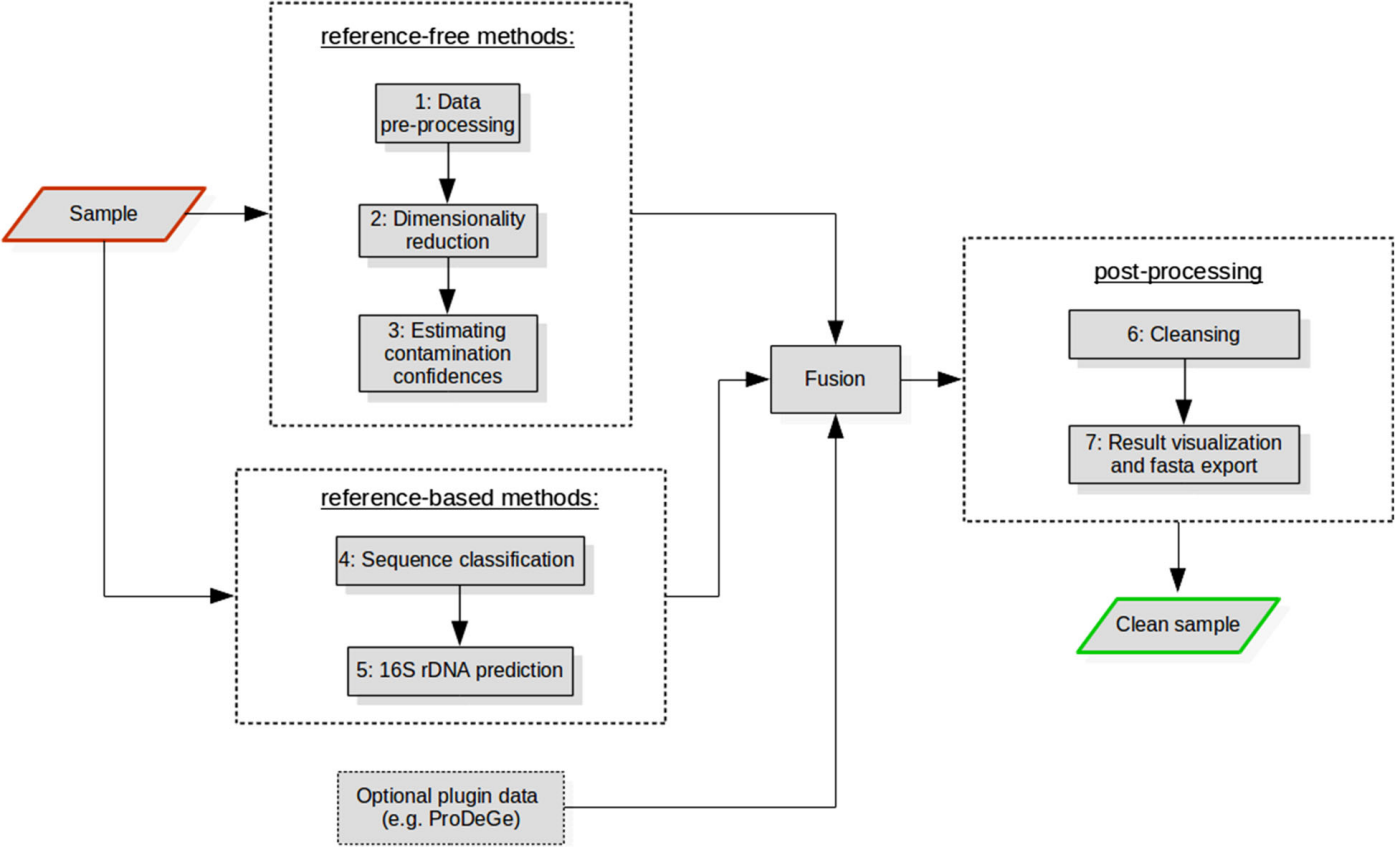
Acdc contamination detection pipeline: Results from both reference-free and reference-based techniques are fusioned and post-processed to end up with a clean sample

Acdc detects contamination in a series of steps which are depicted in Fig. 1. Starting with contigs from a given single-cell genome assembly, both reference-free and reference-based methods are employed. In the former, tetramer frequencies are calculated first (1), resulting in a high-dimensional vectorial representation which makes it possible to apply suitable machine learning algorithms. As its high dimensionality would introduce a number of adverse side effects in further processing, it is crucial to reduce dimensionality (2). This enables the accurate estimation of contamination confidences on the basis of clustering (3). External tools are then used to both classify sequences using ultrafast exact alignment (4) and to predict 16S rRNA genes (5). In the case of detected contamination, further clustering algorithms are employed to enable decontamination and export of clean samples (6). Results are then interactively visualized using a flexible web interface (7). Most of these steps include a number of hyper-parameters crucial in machine learning, for which acdc provides an auto-selection mode with well-tested default values (Table 1). In the future, the integration of results from existing tools such as ProDeGe will help to increase detection performance.

**Table 1:** Description of parameters for various techniques used in acdc

Method	Parameter description
Data pre-processing	Given a target of *n* data points (by default, *n*=1000), the window width is fixed as $w = \sum _{i} l_{i} / n$, where *l*_*i*_ is the length of contig *i*. Default choices of *Δ* *w*=*w*/2 and *k*=4 (tetramer frequencies) are robust. For contigs with *l*_*i*_<*w*, the window width is taken as large as possible (*w*=*l*_*i*_).
BH-SNE	The parameter *θ*=0.5 is a trade-off between speed and accuracy. We set the perplexity perp(*n*)=⌊log(*n*)^2^⌋. It can be seen as an effective neighborhood size that controls the graininess of clusters. A small number of data points *n* receives a small perplexity whereas with growing *n* the perplexity saturates.
DIP	The significance level which is uncritical as it is *α*=0 in the large majority of significant cases. Furthermore, the DIP split threshold, i.e. the percentage of data points, for which multimodality was detected, can be seen as a control of detection precision. We found a default value of *t*_*dip*_=0.001 to work very well throughout all tested data sets.
CC	The number of clusters found depends on the underlying graph. In acdc, the graph is constructed by connecting each data point to it’s *k*_*cc*_ mutual nearest neighbors. The parameter *k*_*cc*_ can be interpreted as the minimum number of data points contained in a separate cluster. To be able to detect also very small contamination, we use a default value of *k*_*cc*_=9.
Bootstrapping	We set the number of bootstraps *B*=10. Setting *B* to a larger number will result in more accurate confidence estimations at the cost of a longer runtime.
Kraken	The only parameter required by Kraken is the database to be used. It can be specified as a parameter to acdc as well.
RNAmmer	16S rRNA gene sequence prediction using RNAmmer does not require any parameters.

### Reference-free detection

#### 1. Data pre-processing

**Fig. 2:**
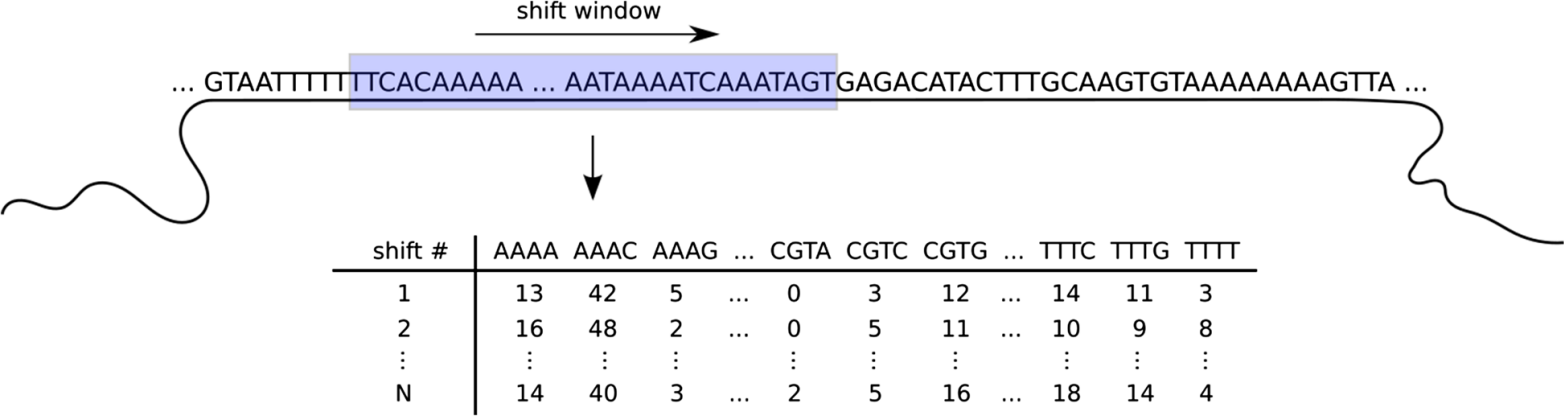
Data pre-processing that transforms a sequential data representation into vectorial data using a sliding window technique: Exemplary for *k*=4, on each shift, a 256-dimensional vector is generated by counting all permutations of the four bases

In order to apply machine learning techniques, it is necessary to transform contigs, represented as sequences, into a vectorial representation. Here, it is common practice to use oligonucleotide signatures [27]. A window of width *w* is fixed and subsequently shifted over the contig sequence using step *Δ* *w* (Fig. 2). For each shift, the underlying *k*-mer frequencies are evaluated. This results in one 4^*k*^-dimensional data point per shift, accounting for the 4 nucleotide bases. For example, taking *k*=4 (tetramers) would result in 256 dimensions, however, by accounting for reverse complements, it can be reduced to 136 dimensions. It is worth noting that taking *k*=1 corresponds to the GC content. The choice of window parameters has considerable influence on the resulting representation. Here, choosing a large window width, capturing genome-specific, rather than gene-specific information will result in less noise [19]. However, a small number of data points is disadvantageous for clustering, such that is has to be taken care to choose *w* not too large. Acdc automatically adjusts window parameters such that for large contigs *w* is homogeneous and for small contigs *w* is adapted to it’s length, i.e. no contigs are discarded. Table 1 includes further information on the setting of *w*. Besides using *k*-mers as a characteristic genomic signature, we looked into using coverage, too. However, due to the coverage bias in multiple displacement amplification [8], using this data for single genomes is problematic.

#### 2. Dimensionality reduction

In machine learning, the analysis of high-dimensional data is severely limited by the so-called curse of dimensionality [26]. To be able to accurately cluster tetramer frequencies, it is crucial to reduce data dimensionality while keeping desired properties such as cluster structure. For this task, modern non-linear dimensionality reduction (t-Distributed Stochastic Neighbor Embedding, t-SNE [28]) and its recent, efficient Barnes-Hut approximation (BH-SNE [22]) is employed. It puts a particular focus on the formation of cluster structures, which enables further clustering algorithms to deliver accurate results. Both qualitative and quantitative analysis [18, 19, 29] of BH-SNE have shown it is superior to both PCA [30] and to using raw high-dimensional vectors, when applied to tetramer frequencies.

#### 3. Estimation of contamination confidences

An integral part of acdc is the confidence and decision of whether a sample is contaminated or not. This problem can be seen as a clustering task. Optimally, one operational taxonomic unit (OTU, the set of genomic sequences from one single cell) is represented as one cluster, implying that the presence of more than one cluster indicates contamination. Thus, the task is to estimate the number of clusters *k*. This requires careful selection of methods and parameters [31]. In contrast to other applications such as metagenomic binning [19], one is not primarily interested in the actual number of clusters, rather in the distinction between *k*=1 (no structure, clean sample) and *k*>1 (clusters, contaminated sample). As the notion of a cluster is ill-posed, this is an inherently difficult task: Most techniques for estimating *k* operate on cluster-specific characteristics, defined for *k*>1 only, making them inapplicable in our case. The case *k*=1 requires an appropriate null model to which the data is compared to in order to be able to detect no structure. We reviewed techniques for this task in the context of contamination detection [32] and found two particularly promising approaches:

1. The dip-statistic test for multimodality of pair-wise distances (DIP), where a significant multimodal distribution indicates *k*>1 [33, 34].
2. Counting the number of strongly connected components (clusters) in a *k*_*cc*_-nearest-neighbor graph (CC). [35]

**Fig. 3:**
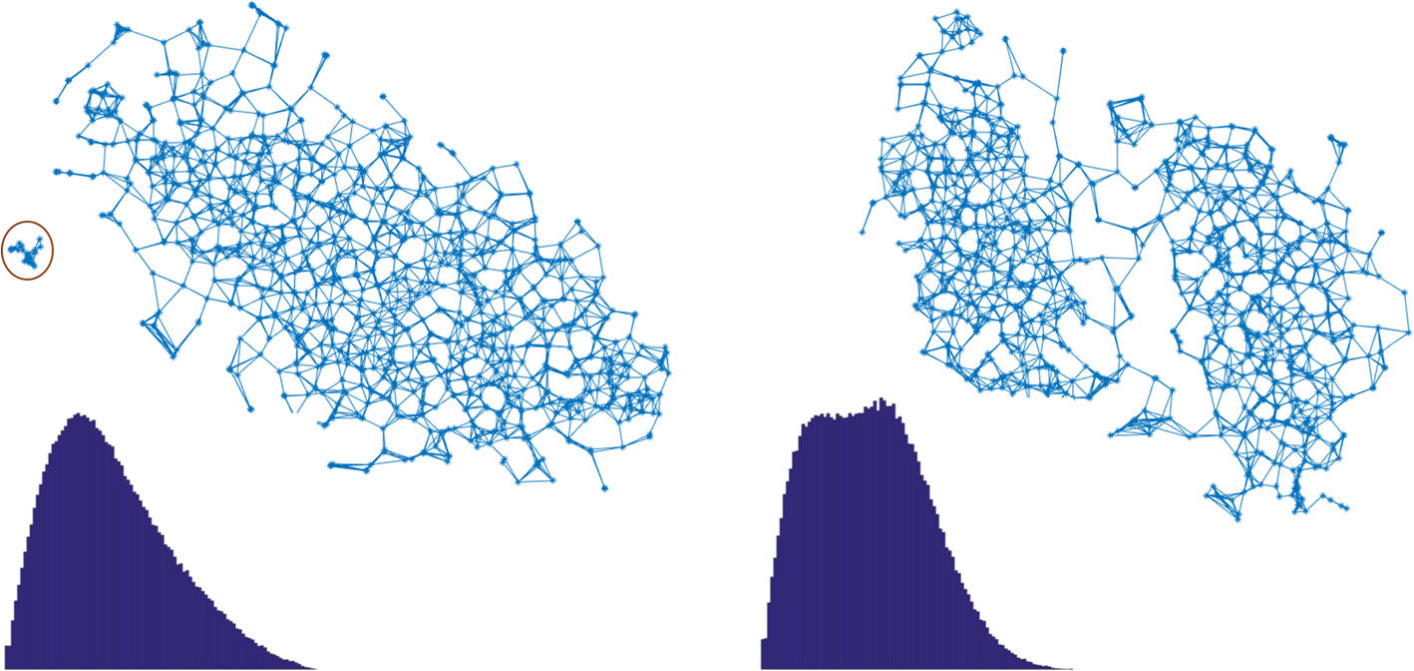
Illustration of the complementary detection capabilities of DIP and CC using two different contaminated samples. *Left*: Using a mutual 9-nearest-neighbor graph, CC identifies two clusters (very small contamination) while DIP isn’t able to detect multimodality as seen in the distribution of pairwise distances below. *Right*: Two overlapping clusters prevent CC from detecting two components while DIP detects significant multimodality in the distribution of pairwise distances

Contamination may occur in a variety of different cluster shapes and sizes. Both methods have been chosen to be employed in acdc to detect those in an antagonistic fashion. While the former is able to detect large and possibly overlapping clusters, the latter is able to detect small and outlier clusters (Fig. 3). Consequently, a given genome assembly is marked as contaminated when DIP or CC indicate more than one cluster.

Furthermore, noisy data, e.g. from very short contigs or from the inherent structure of some species might form separate clusters even in the presence of only one OTU. To prevent false positive contaminant identification from wrongly formed clusters, acdc post-processes cluster assignments in two steps:

1. Disregarding the possibility of chimeric contigs, a contig is expected to appear in only one OTU. Thus, data points that occur in different clusters, but belong to the same contig, indicate a wrong clustering. All points of such a contig are reassigned to the cluster which has the most points of the contig assigned.
2. We include an *aggressive threshold* that determines the minimum number of base pairs that is allowed to form a separate cluster. Smaller clusters are considered as outliers and are neither included into the calculation of contamination confidences nor into cleansing. The default threshold of 5000 *bp* works well throughout all tested data sets. A lower threshold provides more sensitive results towards very low levels of contamination and can be adapted by the user easily.

Last, the machine learning techniques used in acdc, namely dimensionality reduction and clustering, depend on data statistics and hence introduce certain amounts of variance over different runs. In the case of clear contamination, i.e. well separated and compact clusters, these techniques agree with high probability. The same holds true for the case of a clean sample and one well-shaped cluster. However, in the case of an unclear contamination state such as strongly overlapping clusters, results may vary. Hence, it is desirable to provide confidence values gathered over different runs. For this task, acdc employs bootstrapping [26] with which it is possible to calculate statistically substantiated and interpretable confidence measures. We generate bootstraps by randomly sub-sampling 75 percent of the original high-dimensional tetramer data *B*-fold. Each fold is processed by applying dimensionality reduction with BH-SNE and subsequent testing using DIP/CC. A contamination confidence value is obtained by counting the percentage of folds which detected contamination.

### Reference-based detection

#### 4. Sequence classification

We employ Kraken [16] as a fast alternative to the popular BLAST method [12]. Based on a pre-built database, Kraken assigns taxonomic labels to each contig from a sample. Through the use of exact alignments of *l*-mers, it achieves classification accuracy comparable to BLAST while being much faster. In acdc, Kraken classifies contigs on a species level and assigns a taxonomy label to each data point, depending on it’s originating contig. In case of an unclassified species, a contig remains unknown.

Acdc primarily focuses on de novo analysis without existing knowledge from databases and it tackles the challenge to reliably answer the question whether a given sample is contaminated or not. We restricted reference-based cleansing to the fast Kraken method and added an extension for unsupervised detection of potentially non-linear data clusters as performed by acdc. This distinguishes acdc from ProDeGe which relies on both BLASTing predicted genes and a supervised linear separation of contaminants, primarily aiming for an aggressive cleansing with high precision.

#### 5. 16S rRNA gene prediction

Acdc utilizes RNAmmer [36] to predict the location of highly conserved 16S rRNA gene sequences. Even if data could not be classified by Kraken, this enables researchers to identify the higher-level taxonomy of novel species quickly. Additionally, the location of the 16S rRNA gene sequence can be seen as a marker: It is representative for the whole cluster it is located in, and by exporting a clusters (cleansing), the taxonomy for a full OTU can be obtained.

### Post-processing

#### 6. Cleansing

If contamination is detected, acdc finds a clustering which allows the export of contigs from individual clusters, i.e. from the OTU of interest. As this is a process of cleaning the sequence data from unwanted contaminant data, we refer to this as cleansing or decontamination.

For this task, an optimal clustering has to be estimated. While CC provides an optimal assignment by itself, for DIP the number of clusters *k* has to be estimated. In contrast to detecting contamination where the task is to determine either *k*=1 or *k*>1, the cleansing step is slightly different. Similar to metagenomic binning, it is known that *k*>1, which makes it possible to apply methods that estimate the number of clusters using cluster-specific characteristics, only defined for that case. Many clustering and *k*-estimation techniques are available for this task. In [19] it is suggested that the combination of k-means++ as a clustering algorithm and the Davies-Bouldin index [25] as a cluster validity measure works well for binning metagenomic tetramer profiles. In acdc, we replace the k-means++ algorithm by hierarchical clustering using Wards method [37]. We found that it estimates the number of clusters more accurately when there are imbalanced cluster sizes, which we found to be the case in contaminated SCS samples. Therefore, an optimal cluster assignment is determined by finding the minimal (optimal) Davies-Bouldin index for a given range of *k*∈{2,3,4,5}-clusterings using Wards hierarchical clustering.

#### 7. Result visualization

**Fig. 4:**
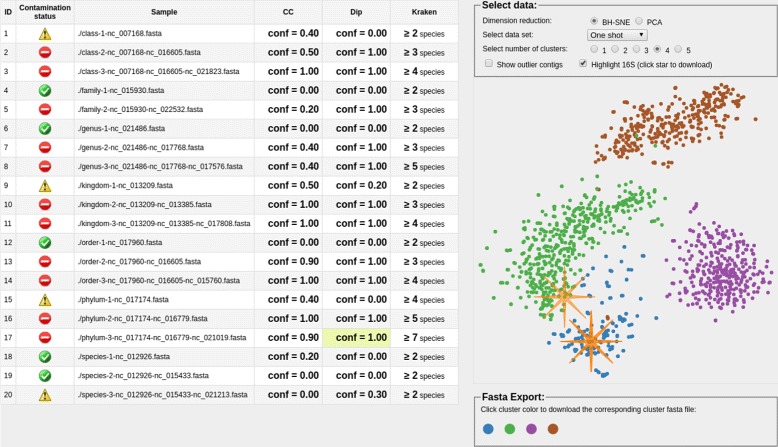
Acdc result interface. For each sample shown in the left-hand side table, visualizations are shown on the right-hand side. Individual clusters can be exported in fasta format by clicking on the respective cluster color on the bottom right

Acdc provides contamination screening results as interactive web pages. An exemplary result of twenty simulated SCS samples is shown in Fig. 4. For each sample on the left hand side, confidences from CC and DIP are shown. A sample is marked clean when for both CC and DIP less than 25 percent of all bootstrap folds found contamination. If either DIP or CC found more than 75 percent of all folds to be contaminated, the sample is marked appropriately. In case of no clear result, a sample is marked with a warning status symbol. A third column with the number of species reported by Kraken is shown. The user is able to inspect each sample for CC, DIP and Kraken. On the right hand side, the sample is visualized using BH-SNE by default. In the event of a wrong cluster assignment, the number of clusters *k* can be selected manually, with the most likely *k* being selected by default. For Kraken, the assignments are fixed and can be inspected by hovering on each data point. Contigs from each cluster can be exported by clicking on the corresponding color in the panel below. Locations of predicted 16S rRNA gene sequences as reported by RNAmmer are indicated by an orange star. A click on it will show the corresponding sequence.

### Computational performance

Acdc has low computational requirements. Input data sizes are in the order of a few megabytes, as we work on assembled contigs, not on the raw data. Given that, using a quad-core consumer laptop, runtimes ranged from a few seconds to ten minutes per sample, depending on the actual size and contamination status. The computationally most expensive step is the calculation of the Dip statistic which has quadratic runtime in it’s worst case and has to be run for all bootstrap folds. This is sped up by parallelization. Memory usage scales linearly with input size.

## Results

The evaluation of our tool can be divided into supervised (database-driven) and unsupervised detection analysis. While the former is restricted to only the method to classify sequences and the size of the underlying database, the latter requires more careful assessment. In order to obtain accurate results, it is necessary to use data with correct ground truth. As the manual assignment of contamination is biased, the simulation of single-cell samples or the analysis of existing samples with references are vital.

To cover a broad range of contaminant varieties, we tested acdc on several simulated and real single-cell sequence data sets:

- **simulated**: We simulated 20 single-cell genomes with varying amounts of contamination and contaminant relatedness. By manually selecting complete genomes from the NCBI database [38], clean and contaminated data sets, each containing up to 3 genomes were generated. Species were chosen such that they are related on different phylogenetic levels, expecting that distantly related species can be better separated than very similar species. For each level, 3 samples were generated, containing 1 – 3 species. The simulation of reads was done using ART [39] followed by subsequent assembly using SPAdes [40].
- **mix**: 9 samples containing 6 draft genomes and 3 single chromosomes were obtained from various sequencing projects (Table 2). All samples are known to be contaminated, however, an exact quantification of contaminated sequences is missing due to the novelty of the data. A detailed description of these data can be found in the Additional file 1.
- **benchmark**: Sequence data from 30 single-cell genomes with low levels of contamination were obtained [41, 42]. Containing cross-contamination between 3 species (*Escherichia coli*, *Meiothermus ruber*, *Pedobacter heparinus*), the median per-sample contaminant proportion of 3% is very small (min=1*%*, max=30*%*).
- **mdm**: Furthermore, 201 single-cell samples from the microbial dark matter (MDM) project [5] were taken to test the capability of our tool on non-contaminated data. These data were manually curated.

**Table 2:** Description and availability of the mix data set. A detailed description of these data can be found in the Additional file 1. Non-available references are denoted by ’NA’

Species name	Ref.	Strain availability
*Herbinix luporum* SD1D^T^	[43]	Prof. Dr. W. Schwarz, Prof. Dr. W. Liebel, Dr. V. Zverlov, Dr. D. Koeck, Technische Universität München, Institute for Microbiology, Munich, Germany
*Clostridium* sp. hoe 37/3	NA	
*Propionispora* sp. 2/2-37	[43]	
*Proteiniborus* sp. DW1	NA	Prof. Dr. H. König, Dr. K.G. Cibis, Johannes Gutenberg-University, Institute for Microbiology and Wine Research, Mainz, Germany
*Peptoniphilaceae* sp. SG1.4B	[44]	
*Methanobacterium formicicum* MF^T^	[45]	
*Methanobacterium formicicum* Mb9	NA	
*Sporanaerobacter* sp. PP17-6a	NA	Dr. M. Klocke and Dr. S. Hahnke, Leibniz-Institut für Agrartechnik Potsdam-Bornim e.V. (ATB), Department of Bioengineering, Potsdam, Germany
*Methanobacterium bourgensis* HAW	NA	Prof. Dr. Scherer, Dr. S. Off, Dr. Y.S. Kim, University of Applied Sciences Hamburg (HAW), Faculty Life Sciences/Research Center ’Biomass Utilization Hamburg’, Hamburg, Germany

We compared acdc to the state-of-the-art contamination detection tool ProDeGe [11] both in terms of supervised and unsupervised detection capabilities. ProDeGe has been optimized to obtain a high precision in the context of a known taxonomic level and database support. It integrates a linear classification model to extend predicted genes to all *k*-mers, displaying excellent behavior in aggressively curating according samples. Unsupervised inspection is restricted to linear PCA only. In contrast, acdc has been optimized to provide good F-measures (i.e. precision and recall) in curating, and it addresses database independent de novo detection of contamination, thus providing a tool highly complementary to ProDeGe.

### Supervised analysis

Both ProDeGe using the BLAST algorithm and acdc using Kraken with the “MiniKraken DB” were tested on the **simulated** and **benchmark** data sets. These are the only two data sets for which entries for known contaminants existed in both used databases. Both tools showed nearly identical high performance (*F*_1_>0.95) in identifying contaminant sequences and didn’t require any further evaluation.

### Unsupervised analysis

The evaluation of unsupervised detection performance was carried out **a)** by testing the ability to detect the correct contamination state of a given sample, and **b)** by measuring the ability to correctly identify clean and contaminant contigs.

**a)** Acdc correctly identified the majority of both contaminated and clean genome assemblies throughout all data sets (Table 3). This result demonstrates the ability of acdc to single out contaminated versus clean genome assemblies, specifically without any reference to a database in de novo settings. For this part of the evaluation, we could not compare to existing methods because they either do not have the functionality to distinguish clean and contaminated samples (ProDeGe), or operate reference-based only (CheckM). Warnings are sometimes issued for assemblies with unclear contamination state. Here, further inspection often revealed the presence of small outlier clusters throughout a small number of bootstraps. In the rare case of strongly unbalanced and additionally overlapping clusters, acdc is not able to detect contamination because of missing structure in the data. Further, if the contaminant is too related to the target (e.g. different strains from the same species), genomic signatures differ only by a very small percentage of all basepairs, making it impossible for acdc to detect them. Interestingly, mdm samples that have been identified as contaminated display a quite distinct cluster structure. Further manual investigation on a small subset of these samples revealed the presence of true contamination which was not identified during manual curation. Furthermore, the sequence of a bacteriophage was identified. Horizontally transferred genetic material such as from bacteriophages or plasmids often have significantly different genomic signatures. Hence, the found structures highlight biologically interesting phenomena.

**Table 3:** Acdc evaluation of contamination detection performance. Entries depict the number of correctly identified clean and contaminated samples with additional information about false predictions in parentheses

Data set	Identified clean samples	Identified contaminated samples
simulated	4/7 (3 warnings)	10/11 (1 warning)
mix	0/0	8/9 (1 warning)
benchmark	0/0	22/30 (6 warnings, 2 clean)
mdm	150/201 (39 warnings, 12 contaminated)	0/0

**b)** We compared^1^ acdc to ProDeGe in terms of precision/recall performance with respect to the number of correctly identified clean basepairs in each sample. For this task, the functionality to export clean sequences common in both tools was used. Since the evaluation is performed for the setting of limited prior biological information, no taxonomy is provided for ProDeGe, restricting the use of reference sequences from databases. Results in Table 4 were averaged over different samples from the simulated and benchmark data sets. Both ProDeGe and acdc correctly identified clean contigs in the benchmark data set with high precision. However, on average acdc was able to recall 22% more clean sequences on the data set, due to the more aggressive design of ProDeGe. Next, ProDeGe was not able to identify the majority of clean sequences in the simulated data set without taxonomic information. In those cases, mostly all contigs were marked as contaminants, resulting in an empty clean sequences file. This fact can be attributed both to ProDeGes behavior of selecting contaminants with high specificity [11] and to it’s missing ability to distinguish between clean and contaminated samples. Results of 4 samples could not be obtained, because computation didn’t provide any output. On the same data, acdc was able to correctly identify the majority of clean sequences with high precision and recall. For samples that contain closely related species, it is difficult to split clean and contaminated sequences. For example, in our simulated data, samples from the same genus contain species with an average nucleotide identity (ANI) of 73%. This fact led to a slight drop in performance. Sequences containing strains from the same species (ANI in our simulated samples: 95%) didn’t contain enough distinct information to be correctly identified, showing the limits of acdc’s reference-free detection capabilities.

**Table 4:** Precision, recall and *F*_1_-scores of predicted clean base pairs for both ProDeGe and acdc on the **simulated** and **benchmark** data sets

Data set	*Precision*	*Recall*	*F*_1_	*Precision*	*Recall*	*F*_1_
	ProDeGe	ProDeGe	ProDeGe	acdc	acdc	acdc
simulated (kingdom)	No result	No result	No result	1.00	1.00	**1.00**
simulated (phylum)	No result	No result	No result	0.99	0.98	**0.99**
simulated (class)	No result	No result	No result	1.00	0.99	**0.99**
simulated (order)	No result	No result	No result	0.99	0.98	**0.99**
simulated (family)	No result	No result	No result	1.00	1.00	**1.00**
simulated (genus)	0.22	0.32	0.22	0.95	0.97	**0.96**
simulated (species)	0.50	0.33	0.36	0.38	0.77	**0.46**
benchmark (*E.coli*)	1.00	0.88	0.93	0.97	0.99	**0.98**
benchmark (*M.ruber*)	1.00	0.73	0.83	0.99	0.99	**0.99**
benchmark (*P.heparinus*)	1.00	0.70	0.81	1.00	1.00	**1.00**

Each row contains average values of the given sub data set. Bold values depict the best performing entry. Entries marked as “no result” either produced an empty clean fasta file or did not finish computation

## Conclusions

Operating both in the presence and absence of references from databases, acdc was able to predict the contamination state in the large majority of samples from four unrelated data sets, containing a total of 258 single-cell genome assemblies. Additionally, clean and contaminant sequences were correctly identified with high recall and precision. In the absence of a given target taxonomy which is required by similar methods (i.e. ProDeGe), acdc was still able to correctly predict contamination based on state-of-the-art techniques from unsupervised machine learning. Complementary to other tools, our software does neither require the prediction of (marker) genes nor existing knowledge from databases to detect contaminants and to separate contaminant from clean sequences. Although, supplemental database information will aid identification, for example of closely related species. These findings make acdc an ideal tool to complement state-of-the-art contaminant detection and cleansing methods such as ProDeGe or CheckM in the context of de novo analysis with limited taxonomic information or limited availability of reference sequence information. Last, as contamination detection and metagenomic binning are closely related, we look forward to applying a modified version of our pipeline to this type of data in the near future.

## Availability and requirements

- **Project name:** acdc
- **Project home page:** https://github.com/mlux86/acdc
- **Operating system:** Linux
- **Programming language:** C++11
- **Other requirements:** None
- **Licence:** MIT

## Endnote

^1^ For the comparison the ProDeGe online version at https://prodege.jgi.doe.gov/was used.

## Additional file

Additional file 1 A detailed description of the **mix** data set. (DOCX 12.8 kb)

## Abbreviations

acdc: Automated Contamination Detection and Confidence estimation for single-cell genome data
ANI: Average nucleotide identity
BH-SNE: Barnes-Hut-SNE
CC: Connected components
DIP: Dip-statistic test for multimodality
MDM: Microbial dark matter
OTU: Operational taxonomic unit
PCA: Principal component analysis
SCS: Single-cell sequencing
t-SNE: The-Distributed Stochastic Neighbor Embedding
