# Supplementary material for: acdc – Automated Contamination Detection and Confidence estimation for single-cell genome data
Source: BMC Bioinformatics. 2016 Dec 20;17:543. doi: 10.1186/s12859-016-1397-7 (PMC5168860; doi:10.1186/s12859-016-1397-7)
Supplement: Additional file 1 — A detailed description of the mix data set. (DOCX 12.8 kb) [file 12859_2016_1397_MOESM1_ESM.docx]

# Detailed description of the 'mix’ data set

***Herbinix luporum* SD1D^T^**

*Herbinix luporum* SD1D^T^ was isolated from a thermophilic biogas plant (Maus *et al*.; Koeck *et al*., 2016) and represents a novel species within the genus *Herbinix* (family *Lachnospiraceae*) (Koeck *et al.,* 2016). This cellulolytic bacterium is able to degrade various carbon sources (filter paper, phosphoric acid swollen cellulose (PASC), xylan, galactose, mannose) and produces ethanol, acetic and propionic acid.

Koeck DE, Maus I, Wibberg D, Winkler A, Zverlov VV, Liebl W, Pühler A, Schwarz WH, Schlüter A. (2016). Complete genome sequence of *Herbinix* *luporum* SD1D^T^, a new cellulose degrading bacterium isolated from a thermophilic biogas reactor. Genome Announc. In press.

Maus I, Koeck DE, Cibis KG, Hahnke S, Kim YS, Langer T, Kreubel Y, Erhard M, Bremges A, Off S, Stolze Y, Jaenicke S, Goesmann A, Sczyrba A, Scherer P, König H, Schwarz WH, Zverlov VV, Liebl W, Pühler A, Schlüter A, Klocke M. Unraveling the microbiome of a thermophilic biogas plant by metagenome and metatranscriptome analysis complemented by characterization of bacterial and archaeal isolates. Biotechnol Biofuels. 2016 Aug 11;9:171

***Clostridium* sp. Hoe 37/3**

A novel strain, namely *Clostridium* sp. hoe 37/3, was obtained from a mesophilic agricultural biogas plant utilizing maize and grass silage for biomethanation. This mesophilic bacterium is able to utilize crystalline cellulose for growth; butyrate and ethanol are the end-products of the carbohydrate fermentation (unpublished data).

***Propionispora* sp. 2/2-37**

The strain *Propionispora* sp. 2/2-37 was isolated from an industrial mesophilic one-phase biogas reactor digesting a mixture of maize silage, wheat straw and manure (Koeck et al., 2016). The obtained isolate belongs to the genus *Propionispora* within the order *Selenomonadales* (class *Negativicutes*). The 2/2-37 genome possesses genes encoding enzymes facilitating growth on a great variety of mono-, di- and polysaccharides including cellobiose, sorbitol and xylooligosaccharide, indicating its involvement in acidogenesis in the course of anaerobic digestion.

Koeck DE, Maus I, Wibberg D, Winkler A, Zverlov VV, Liebl W, Pühler A, Schwarz WH, Schlüter A. (2016). Draft genome sequence of *Propionispora* sp. 2/2 37, a new mesophilic xylan degrading bacterium isolated from a mesophilic biogas reactor. Genome Announc. 2016 Jun 23;4(3).

***Proteiniborus* sp. DW1**

The mesophilic bacterium *Proteiniborus* sp. DW1 was originally isolated in co-culture with *Methanobacterium* sp. Mb1 (Maus et al., 2013). The culture originated from a rural biogas plant producing methane-rich biogas from maize silage and cattle manure in Germany. Genome analysis revealed that the strain DW1 possesses genes involved in the utilization of different carbohydrates. Lactate is assumed to be required for propionic acid production (unpublished data).

Maus I, Wibberg D, Stantscheff R, Cibis K, Eikmeyer FG, König H, Pühler A, Schlüter A. (2013). Complete genome sequence of the hydrogenotrophic *Archaeon* *Methanobacterium* sp. Mb1 isolated from a production-scale biogas plant. J Biotechnol. 168(4):734-6.

***Peptoniphilaceae* sp. SG1.4B**

The bacterial strain SG1.4B of the family *Peptoniphilaceae* (Cibis *et al*., 2016) was isolated from a thermophilic full-scale biogas plant fed with maize and grass silage and pig manure as substrates for biomethanation. The obtained isolate belongs to the family *Peptoniphilaceae* (order *Clostridiales*) showing 89% 16S rRNA gene sequence similarity to Gallicola barnesae DSM-3244 (Cibis *et al*., 2016).

Cibis KG, Gneipel A, König H. (2016). Isolation of acetic, propionic and butyric acid-forming bacteria from biogas plants. J Biotechnol. 220:51-63.

***Methanobacterium formicicum* MF^T^**

The neotype strain *Methanobacterium formicicum* MF^T^ (Maus et al., 2014) was originally obtained from a domestic sewage sludge digestor in Urbana (Illinois, USA) (Sousa et al., 2007; Bryant and Boone, 1987). It produces methane from H_2_, CO_2_ and formate. For sequencing purposes, the strain MF^T^ was obtained from the DSMZ (Leibniz Institute German Collection of Microorganisms and Cell Cultures, Braunschweig, Germany).

Maus I, Stantscheff R, Wibberg D, Stolze Y, Winkler A, Pühler A, König H, Schlüter A. (2014). Complete genome sequence of the methanogenic neotype strain Methanobacterium formicicum MF(T.). J Biotechnol. 192 Pt A:40-1.

***Methanobacterium formicicum* Mb9**

The strain *Methanobacterium formicicum* Mb9 was isolated from a fermentation sample of a rural, mesophilic, industrial-scale biogas plant fed with maize silage, grass silage and liquid bovine manure. As hydrogenotrophic methanogen, *M. formicicum* Mb9 contains genes encoding the complete methanogenesis pathway using H_2_ and CO_2_ as substrates.

Tejerizo GT, Kim YK, Maus I, Wibberg D, Off S, Pühler A, Scherer P, Schlüter A. (submitted). Genome sequence of *Methanobacterium curvum* strain Buetzberg, a hydrogenotrophic, methanogenic archaeon, isolated from a mesophilic industrial-scale biogas plant utilizing bio-waste. J Biotechnology.

***Sporanaerobacter* sp. PP17-6a**

A novel bacterial strain, namely *Sporanaerobacter* sp. PP17-6a, was isolated from an inoculum obtained from a mesophilic lab-scale completely stirred tank reactor (CSTR) system continuously fed with a mixture of maize silage, pig and cattle manure.

Sousa, D.Z., Smidt, H., Alves, M.M., Stams, A.J., 2007. *Syntrophomonas zehnderi* sp. nov., an anaerobe that degrades long-chain fatty acids in co-culture with *Methanobacterium formicicum*. Int J Syst Evol Microbiol. 57(Pt 3):609-15.

Bryant, M.P. and Boone, D.R., 1987. Isolation and characterization of *Methanobacterium forrnicicum* MF. Appl Environ Microbiol. 54(3):693-8.

***Methanoculleus bourgensis* HAW**

The methanogenic Archaea *Methanoculleus bourgensis* strain HAW was isolated from a mesophilic full-scale biogas fermenter operating under high ammonium concentration and utilizing bio-waste for anaerobic digestion. *M. bourgensis* HAW is predicted to utilize H_2_ and CO_2_ for methane synthesis, since all genes involved in methanogenesis were identified in the HAW genome. Acetate is predicted to be required for growth (unpublished data).
